# CRISPR/Cas9 Reduces Viral Load in a BALB/c Mouse Model of Ocular Herpes Infection

**DOI:** 10.3390/biomedicines13071738

**Published:** 2025-07-16

**Authors:** Luiza Silveira Garcia, Rafaela Moraes Pereira de Sousa, Viviane Souza Campos, Erik Machado Ferreira, Cynthia Machado Cascabulho, Elen Mello de Souza, Vanessa Salete de Paula

**Affiliations:** 1 Laboratório de Virologia e Parasitologia Molecular, Instituto Oswaldo Cruz/FIOCRUZ, Rio de Janeiro 21040900, RJ, Brazil; luizagarcia@aluno.fiocruz.br (L.S.G.); rafaelamoraes@id.uff.br (R.M.P.d.S.); 2 Laboratório de Biologia Estrutural, Instituto Oswaldo Cruz/FIOCRUZ, Rio de Janeiro 21040900, RJ, Brazil; vivianecs@id.uff.br; 3 Laboratório de Hepatites Virais, Instituto Oswaldo Cruz/FIOCRUZ, Rio de Janeiro 21040900, RJ, Brazil; erikmafer@gmail.com; 4 Plataforma de Citometria de Fluxo-Unidade de Análise Multiparamétrica, Instituto Oswaldo Cruz/FIOCRUZ, Rio de Janeiro 21040900, RJ, Brazil; cynthiac@ioc.fiocruz.br

**Keywords:** herpetic keratitis, CRISPR/Cas9, in vivo model, HSV-1

## Abstract

**Background/Objectives**: *Simplexvirus humanalpha1* (HSV-1) can cause herpetic keratitis, which is the most common cause of infectious blindness in developed countries. Some patients can develop toxicity or resistance to available treatments and may require keratoplasty. **Methods:** As an alternative therapy, the CRISPR/Cas9 anti-HSV-1 activity was assessed in an experimental model of BALB/c mice. **Results:** The results showed that the viral load in the eyes of mice inoculated with HSV-1 at 10^7^ PFU/mL was 4.5 ± 0.2 log_10_ copies/mL. In contrast, mice inoculated with 10^9^ PFU/mL exhibited a high viral load of 8.1 ± 0.4 log_10_ copies/mL. The detection of HSV-1 DNA and lesions in the eye was consistent with the viral inoculum of the infection. Next, antiviral activity showed that 200 ng/µL of CRISPR/Cas9 reduced the viral load by 2 logs (*p* ≤ 0.0001), as well as the lesion scores, compared to the untreated group. **Conclusions:** Together, the data suggest that CRISPR/Cas9 could be investigated as an alternative therapy for ocular herpes.

## 1. Introduction

*Simplexvirus humanalpha1* (HSV-1) is one of the most prevalent human viruses, affecting approximately 67% of the world’s population [1]. Clinical symptoms of ocular viral infection include myalgia, dryness, conjunctivitis, blepharitis, and corneal damage. HSV-1 can cause latent infections in sensory neurons after primary infection and replication in the corneal epithelium. It is retrogradely transported through ophthalmic nerves to the trigeminal ganglia (TG), where it establishes a lifelong latent reservoir [2]. Viral replication can be reactivated under certain conditions, such as immunosuppression, organ transplantation, ultraviolet irradiation, stress, and coinfections, leading to ocular disease recrudescence [3]. Herpes simplex keratitis (HSK) is a typical vision-threatening condition characterized by recurrent corneal infections in which the virus is often not detected [4]. Estimates show that there are 1.5 million HSV ocular episodes annually, and 40 thousand cases of visual impairment are reported worldwide, with corneal transplantation often required [4,5]. Even when transplantation is performed, the likelihood of rejection cannot be disregarded. A study based on a 5-year follow-up showed that 21% of recipients experienced at least one endothelial rejection episode [6]. The rate of patients experiencing rejection after corneal transplantation ranges from 13.6% to 29.2% in Brazil alone [7].

HSV-1 infection is treated with purine nucleoside analogs, such as guanosine. Acyclovir, which was developed almost 50 years ago, is the first-line medication for these cases, followed by its analogs valaciclovir and famciclovir, all of which target viral DNA polymerase [8]. Continuous acyclovir administration has led to drug resistance development in 6.4% of cases [9]. Studies have shown that specific groups, including immunocompromised patients and individuals subjected to chronic antiviral therapy, can present deletion, insertion, or substitution of nucleotides in the UL23 and UL30 genes, which are involved in resistance development [10,11]. Acyclovir is primarily excreted by the kidneys and, in some cases, can cause renal injuries, as well as neurotoxicity [12].

Clinical and preclinical studies have investigated the use of the gene-editing technology known as CRISPR/Cas9. The Food and Drug Administration (FDA) has approved CRISPR/Cas9 for phase I/II trials to treat the following genetic diseases: β-thalassemia, sickle cell disease, and Leber congenital amaurosis (LCA-10/ retinal disease) (ClinicalTrials.gov: NCT04208529; NCT03745287; NCT03872479, respectively) [13]. The therapeutic potential of CRISPR/Cas9 for treating infectious diseases has been supported by its demonstrated antimicrobial [14], antiparasitic [15], and antifungal efficacy [16]. For viruses, studies addressing human immunodeficiency virus (HIV) treatment are more advanced [17,18,19,20]. Studies of the antiviral activity of CRISPR/Cas9 in vitro and in vivo have been investigating herpes simplex virus, Epstein–Barr virus, cytomegalovirus, and Kaposi’s sarcoma-associated herpesvirus [3,21,22,23].

The main aim of the current preclinical study was to assess the antiviral effect of CRISPR/Cas9 in a BALB/c mouse model infected with a Brazilian HSV-1 strain. It was observed that high viral-load infection causes herpetic keratitis in the non-scarified corneas of mice and that a single CRISPR/Cas9 application can be sufficient to reduce both the viral load and clinical signals in mice during the kinetic infection.

## 2. Materials and Methods

### 2.1. Virus

The Brazilian HSV-1 strain (GenBank accession number: JQ673480.1; NCBI: txid10306), isolated from a human case of recurrent oral herpes with blisters, was used for ocular infections in BALB/c mice [24]. VERO cell line cultures were used to propagate and titrate the virus [25], which was stored at –70 °C until the assay.

### 2.2. Animals

BALB/c male mice (6–8 weeks) were provided by the Institute of Science and Technology in Biomodels (ICTB/FIOCRUZ, Rio de Janeiro, Brazil). Five mice were housed in each cage. They were kept in a conventional room at 20–25 °C under a 12/12 h light/dark cycle. Mice were left to acclimate for 7 days before the experiments and had access to sterilized food and water *ad libitum*.

All assays were run in triplicate, and samples were analyzed three days after infection. The procedures were carried out in accordance with the IOC guidelines from the Committee of Ethics for the Use of Animals (CEUA/IOC-040/2019, approved on 10 December 2019).

### 2.3. HSV-1 Infection

The mice were divided into experimental groups. Ocular infection was performed in non-scarified cornea by inoculating 5 μL viral solution in the right eye (10^7^ or 10^9^ PFU/mL). Age-matched non-infected mice (mock) were inoculated with 5 μL phosphate buffered saline (PBS) and kept under identical conditions.

Ten to fifteen mice from each group were analyzed during infection kinetics (4th, 7th, and 14th dpi). This was done to assess infection model establishment and antiviral therapeutic activity.

### 2.4. Viral Load Assessment Through qPCR

HSV-1 DNA absolute quantification was performed through real-time PCR (qPCR). Viral DNA was extracted from mice’s eyes using the DNeasy Tissue Kit (Qiagen Inc, Germantown, MD, USA). Lima and collaborators described the primers, probe target UL-39 genes, and standard curve used for HSV-1 DNA quantification [26]. The qPCR reaction was performed using the AgPath-ID PCR kit (Life Technologies, Carlsbad, CA, USA) which contained 20 μL of a mix comprising 12.5 μL 2× buffer, 2.5 μL primers sense (1 μM), 2.5 μL antisense primers (1 μM), 2 μL specific probe (0.4 μM), and 1 μL 1× enzyme. In addition, qPCR was performed for negative controls (H_2_O DNase and RNase free), and known quantities of the HSV-1 DNA fragment were used to generate the standard curve.

### 2.5. Clinical Manifestation Analyses

Clinical manifestations were assessed through weight curve and periocular/ocular lesion sign parameters.

The body weight of mice was monitored on the 0, 4th, 7th, and 14th dpi. The results were expressed as mean ± standard deviation, and they represented variations among different experimental groups.

Periocular/ocular lesion signs in infected, and untreated or treated groups of mice were followed-up on the 0, 4th, 7th, and 14th dpi. Clinical signs of periocular/ocular lesion were classified through scores ranging from 0 to 4, as follows: absent (0), mild (1), moderate (2), intense (3), and severe (4), according to descriptions in Table 1.

### 2.6. CRISPR/Cas9 Treatment

CRISPR/Cas9 antiviral therapeutic activity was assessed during HSV-1 in vivo infection. The construction of CRISPR/Cas9-mediated genome editing targeting the *UL39* gene of HSV-1 and in vitro testing was previously detailed. The sgRNA sequence was inserted into the plasmid (PX459-UL39) [21]. A supplementary file shows the guide RNA sequences and the UL39 region from HSV-1 (3518 bases pairs) which were selected and tested in a previous study (Table S1 and Figure S1) [21,24].

The animals were divided into six groups: a negative control group (non-infected), two positive control groups (infected with HSV-1 at concentrations of 10^7^ PFU/mL or 10^9^ PFU/mL without treatment), and three treatment groups (infected with HSV-1 at 10^9^ PFU/mL and treated with CRISPR/Cas9 at concentrations of 50, 100, or 200 ng/μL).

On the 1st day post-infection, the groups of mice were treated with a single application of 5 μL of CRISPR/Cas9 targeting HSV-1, at concentrations of 50, 100, or 200 ng/μL. The solution was administered as eye drops, directly into the right eye, allowing the CRISPR/Cas9 to be absorbed through the cornea, without the need for an additional delivery system. The untreated groups received 5 μL of PBS under the same experimental conditions (Table 2).

### 2.7. Statistics

Results recorded for three independent experiments were analyzed in Prism 9.3 software (GraphPad Software Inc., San Diego, CA, USA). All analyses were based on One-Way ANOVA, with Dunnett’s multiple comparisons test, or with one-tailed *t*-test. Significance values were established as * *p* ≤ 0.01, ** *p* ≤ 0.001 and *** *p* ≤ 0.0001.

## 3. Results

### 3.1. Ocular Damage Was Associated with Viral Inoculum During HSV-1 Infection Kinetics

Infection kinetics was carried out to quantify the viral load and to observe the development of periocular/ocular lesion signals. Initially, BALB/c mice inoculated with HSV-1 10^7^ and 10^9^ PFU/mL, directly in the eye, were assessed on the 4th, 7th, and 14th day post infection (dpi).

**Figure 1:**
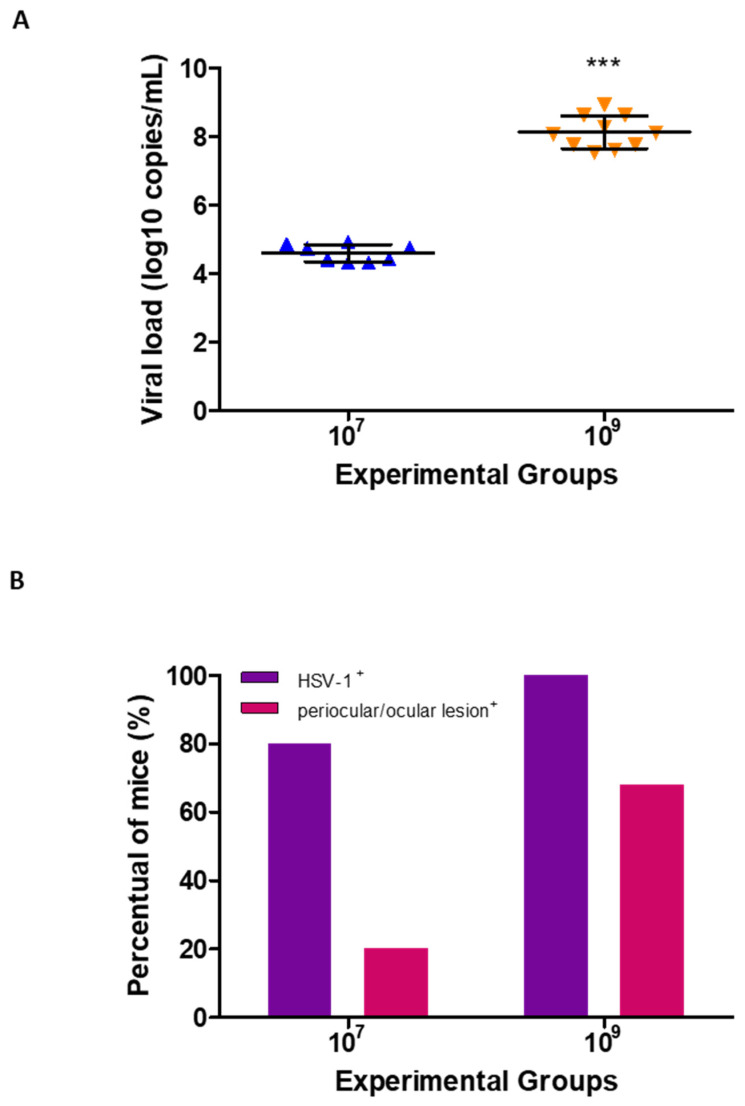
Assessing viral load through qPCR and lesions in the eye of BALB/c mice infected with HSV-1 at 10^7^ and 10^9^ PFU/mL: (**A**) Quantifying HSV-1 DNA copies of mice inoculated with 10^7^ and 10^9^ PFU/mL, on the 4th, 7th, and 14th dpi at different time points throughout the infection. The graph represents the mean ± standard deviation of each experimental group. *** *p* ≤ 0.0001 statistical difference in relation to other. (**B**) HSV-1 DNA detection (positive) and some periocular/ocular lesion signs (positive) in mice inoculated with 10^7^ and 10^9^ PFU/mL. The graph represents the percentages of animals HSV-1 DNA positive on the 4th dpi and of those with ocular lesions on the 7th and 14th dpi, in each experimental group.

HSV-1 DNA was quantified through qPCR, revealing distinct viral load profiles between the infection groups. Mice inoculated with 10^7^ PFU/mL exhibited a consistent viral load of 4.5 ± 0.2 log_10_ copies/mL throughout the infection kinetics. In contrast, mice infected with 10^9^ PFU/mL displayed significantly higher viral loads, reaching 8.1 ± 0.4 log_10_ copies/mL at all time points (Figure 1A).

Quantification of HSV-1 DNA by qPCR also revealed differences in the percentage of infected mice between the groups. By the 4th dpi, the proportion of qPCR-positive mice increased according to the viral inoculum. In the 10^7^ PFU/mL group, 80% of the mice tested positive, while in the 10^9^ PFU/mL group, 100% of the mice were qPCR-positive (Figure 1B).

Similarly, the incidence of periocular/ocular lesions was dose-dependent on both the 7th and 14th dpi. On the 7th dpi, 20% of mice in the 10^7^ PFU/mL group exhibited lesion signs, whereas in the 10^9^ PFU/mL group, this rate reached 68% (Figure 1B). These findings suggest that higher viral loads are associated with increased lesion severity and a higher percentage of affected mice.

**Figure 2:**
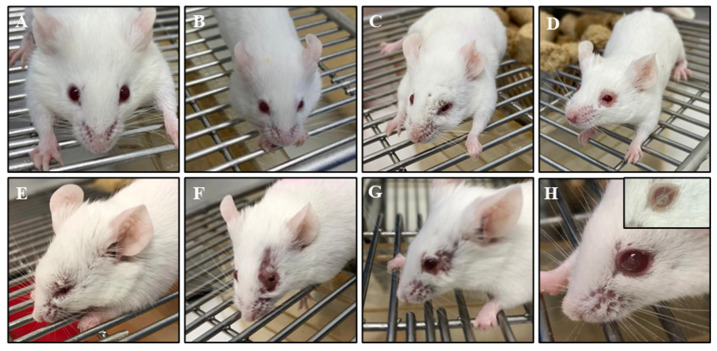
Periocular/ocular lesion scores in BALB/c mice infected with HSV-1. (**A**) Uninfected mice (mock, score 0). (**B**–**D**) Mice infected with 10^7^ PFU/mL showing mild (score 1, (**B**)), moderate (score 2, (**C**)), and intense (score 3, (**D**)) lesions. (**E**–**H**) Mice infected with 10^9^ PFU/mL exhibiting severe lesions (score 4).

The clinical signs of periocular/ocular lesions induced by HSV-1 infection were assessed using a scale ranging from 0 to 4 (Figure 2). Uninfected (mock) mice showed no clinical signs throughout the experiment and served as the control group (Figure 2A). In contrast, HSV-1-infected mice exhibited a range of lesion severities.

Clinical observations revealed that mice inoculated with 10^7^ PFU/mL developed lesions starting from the 7th dpi. In this group, mice exhibited mild facial edema (score 1, Figure 2B) and moderate facial edema with periocular/ocular inflammation (score 2, Figure 2C). Some mice in this group also presented intense facial edema and periocular hair loss, corresponding to score 3 (Figure 2D).

**Figure 3:**
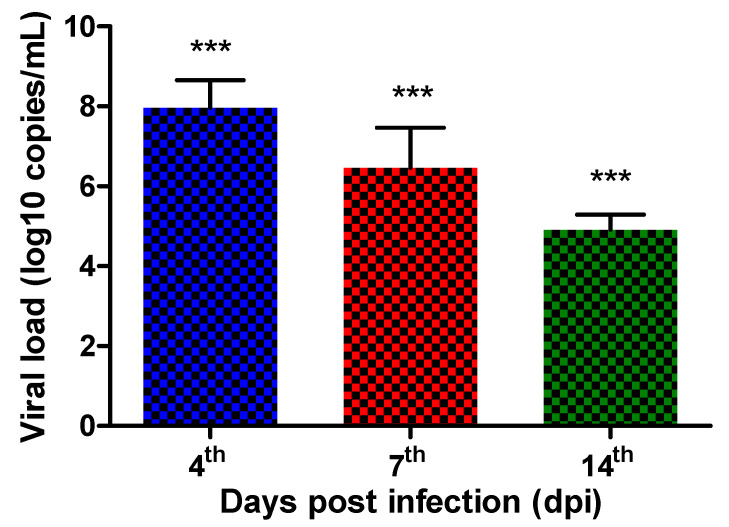
Assessing viral load through qPCR in the eyes of BALB/c mice infected with HSV-1: Quantifying the number of HSV-1 DNA copies during infection kinetics in mice inoculated with 10^9^ PFU/mL. Graph represent the mean ± standard deviation of each day post infection. *** *p* ≤ 0.0001 statistical difference between one and all others.

However, mice infected with 10^9^ PFU/mL began developing lesions as early as the 4th dpi (score 1 and 2), but the severity increased significantly by the 7th dpi (score 1–4). At this stage, affected mice exhibited severe facial edema and periocular/ocular inflammation (Figure 2E), along with extensive hair loss, classified as score 4 (Figure 2F,G). Infection kinetics also indicated a delayed resolution of inflammation, with signs of recovery (scores 1 and 2) observed around the 14th dpi, confirming the self-limiting nature of the infection. However, some mice developed long-term sequelae, such as corneal opacity (Figure 2H, inset). To account for the self-limiting effect, only viral loads from the 4th dpi onward were considered in the infection kinetics analysis (Figure 3).

The self-limiting nature of the periocular/ocular lesions was also reflected in the viral load, which decreased throughout the infection kinetics. However, while mice infected with 10^9^ PFU/mL showed high viral load levels on the 4th dpi (8 ± 0.7 log_10_ copies/mL), these values decreased on the 7th (6.5 ± 1.0 log_10_ copies/mL) and 14th dpi (4.9 ± 0.4 log_10_ copies/mL) (Figure 3). Therefore, the 4th dpi was selected for dose-response curve analysis of the treatment, as it displayed the highest viral load.

### 3.2. CRISPR/Cas9 Treatment Reduced Viral Load and Periocular/Ocular Lesion During HSV-1 Infection Kinetics

**Figure 4:**
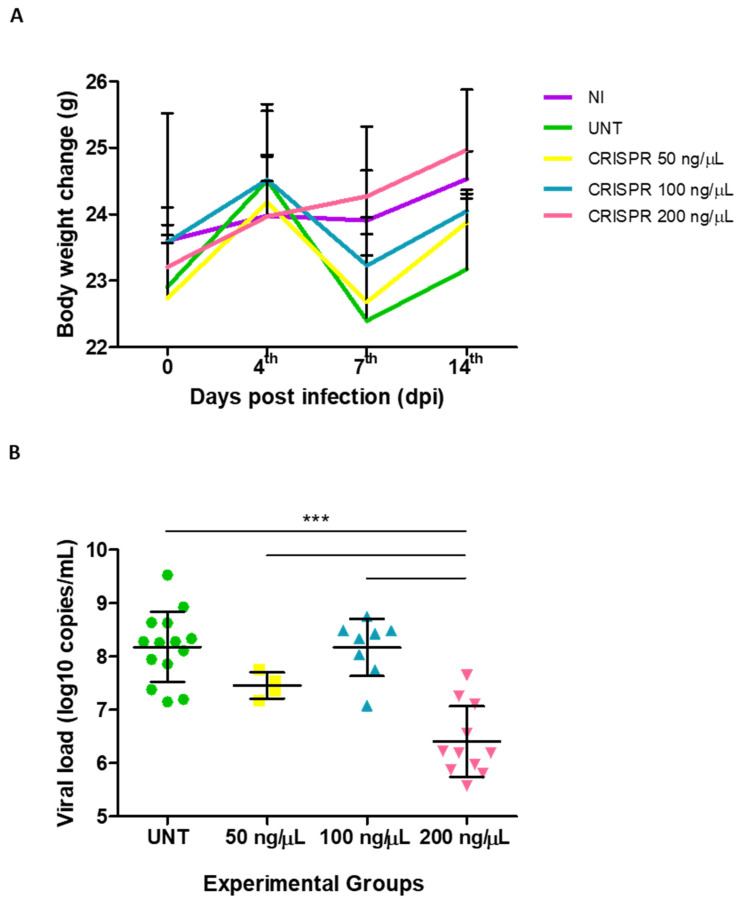
Therapeutic effect of CRISPR/Cas9 on BALB/c mice infected with HSV-1: (**A**) Assessing body weight during treatment with CRISPR/Cas9 (50, 100, 200 ng/µL) compared to the non-infected (NI) and untreated (UNT) groups. The graph represents the mean ± standard deviation of the weight curve plotted for each experimental group during infection kinetics. (**B**) Dose–response curve of CRISPR/Cas9 effect on viral load compared to the untreated group on the 4th dpi, based on qPCR. The graph represents the mean ± standard deviation of each experimental group. *** *p* ≤ 0.0001, indicating a statistically significant difference for the group treated with 200 ng/µL CRISPR/Cas9 compared to all other groups (untreated and treated with 50 and 100 ng/µL).

The anti-HSV-1 therapeutic effect of CRISPR/Cas9 in comparison to the untreated group was assessed. Viral load was quantified in mice infected with 10^9^ PFU/mL, and untreated or treated on the 1st dpi with a single ocular application of 50, 100, or 200 ng/μL CRISPR/Cas9 (Figure 4). Initially, the mice’s body weight during the infection kinetics was not affected by the treatment with different concentrations of CRISPR/Cas9. The results evidenced that mice in all groups showed body weight gain on the 4th dpi; however, weight loss ranging from 0.9 g to 2 g was observed on the 7th dpi, except for mice treated with 200 ng/μL CRISPR/Cas9. Nevertheless, this difference was not statistically significant.

Importantly, all mice had gained weight on the 14th dpi (Figure 4A). This was likely due to the fact that the infection is self-limiting, allowing the mice to gradually recover over time as the viral load decreased and the inflammatory response was controlled.

Consistent with these observations, a single dose of treatment administered on the 1st dpi did not induce toxic effects in vivo, as evidenced by the absence of significant body weight alterations during the acute phase. This aligns with our previous in vitro findings, where no significant cytotoxic effects were observed in edited cells (21)

To further evaluate treatment efficacy, the viral load in the ocular tissue of infected and treated mice was assessed on the 4th dpi (Figure 4B). Data revealed a viral load of 7.5 ± 0.7, 8.2 ± 0.2, and 6.5 ± 0.5 log_10_ copies/mL in groups treated with 50, 100, and 200 ng/μL CRISPR/Cas9, respectively, compared to the untreated group, which exhibited values of 8.2 ± 0.6 log_10_ copies/mL (Figure 4B).

**Figure 5:**
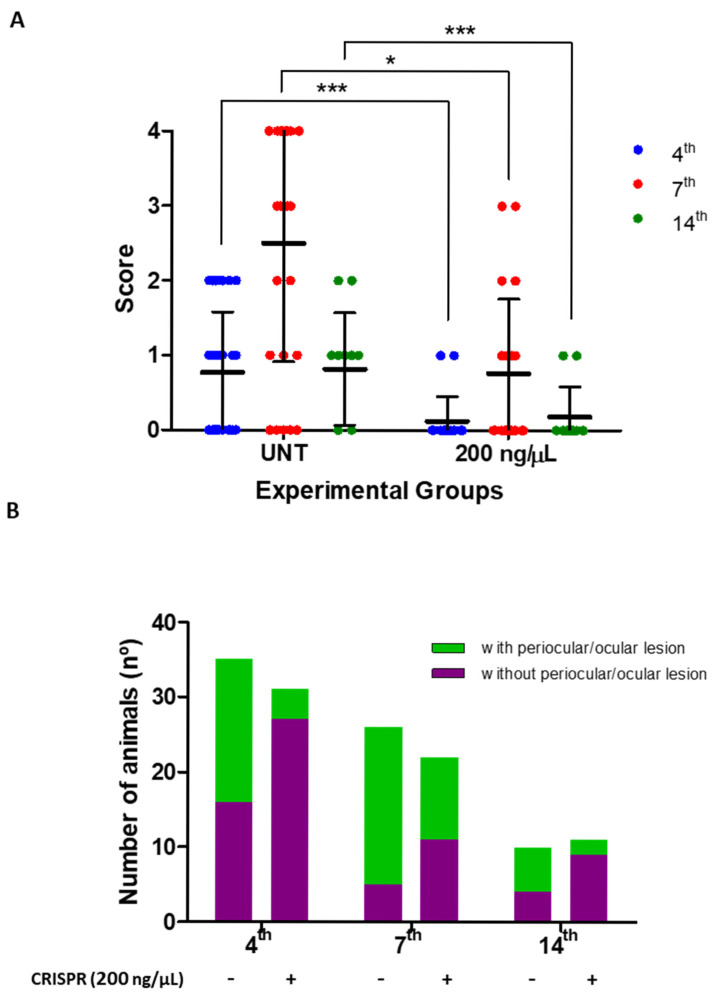
Therapeutic effect of 200 ng/µL CRISPR/Cas9 on periocular/ocular lesion development in BALB/c mice infected with HSV-1: (**A**) score ranging based on periocular/ocular lesion signs in untreated (UNT) and treated (200 ng/µL) groups. The graph represents the mean ± standard deviation of each experimental group during infection kinetics. * p ≤ 0.01 and *** *p* ≤ 0.0001 statistical difference recorded for the group treated with 200 ng/µL CRISPR/Cas9 in relation to the UNT group on each dpi. (**B**) Total number of animals without (purple) or with (green) periocular/ocular lesions in the untreated (-) and treated (+) groups with 200 ng/µL CRISPR/Cas9. The total number of animals reflects the cumulative counting of the three independent experiments during the days post infection.

In addition, the impact of the therapeutic treatment with 200 ng/μL CRISPR/Cas9 on eye damage development was assessed through the scoring criteria described in Table 1 (Figure 5). The infected and untreated group started developing lesions on the 4th dpi (scores 1 and 2) (*p* ≤ 0.0001), with lesions increasing by the 7th dpi (*p* ≤ 0.0001), when mice already had intense and severe clinical signs, i.e., scores 3 and 4 (Figure 5A). However, just like viral load, periocular/ocular lesions also significantly decreased by the 14th dpi (*p* ≤ 0.0001), regressing to scores 1 and 2 (Figure 5A). A CRISPR/Cas9 treatment effect analysis showed significant reduction in scores compared to the untreated group on all infection days. The treated group recorded score 1 on the 4th dpi (*p* ≤ 0.0001), scores 1 to 3 on the 7th dpi (*p* ≤ 0.01), and score 1 on the 14th dpi (*p* ≤ 0.0001) (Figure 5A). The total number of animals from the three experiments that presented any periocular/ocular lesions during all days post infection is also shown (Figure 5B). In the group treated with 200 ng/μL CRISPR/Cas9, only 13% (4th dpi), 50% (7th dpi), and 18% (14th dpi) of mice presented periocular/ocular lesions compared to 45%, 81%, and 60% in the untreated group, respectively (Figure 5B). The treatment led to a 31% reduction in the number of animals showing periocular/ocular lesions on the 7th dpi, when the clinical signs of these lesions were stronger in all groups. This reduction reached 42% in comparison to the untreated group on the 14th dpi, even when self-limiting periocular/ocular lesion had already occurred (Figure 5B).

## 4. Discussion

Infections caused by herpesviruses are among the most widely spread diseases at the global scale. According to data provided by the World Health Organization, more than 3.7 billion people under 50 years are affected by HSV-1 [27]. The present study showed that a high-load HSV-1 inoculum applied as a suspension in eyes without corneal scarification resulted in ocular lesion development and that treatment with 200 ng/µL of CRISPR/Cas9 can reduce viral load and ocular lesions in comparison to the untreated group.

HSV-1 latency, reactivation, and illness recurrence, in addition to toxicity and resistance to antiviral therapies, are important gaps to be investigated. However, experimental models with which to test new prophylactic and therapeutic treatments for herpetic keratitis remain a challenge. Several studies have shown variability between experimental models, since the HSV-1 strain, as well as the infection route and mice lineages and gender, lead to mortality rate, infection severity, and reactivation frequency variables [28,29,30]. In our study, BALB/c mice were used as an infection model, as previous studies have shown that most HSV-1 strains are reactivated faster in BALB/c than in C57BL/6 mice, probably due to their immune background [28,31]. Moreover, we observed clinical manifestations of the herpetic keratitis caused by ocular inoculation with HSV-1 without corneal scarification in BALB/c mice similar to those found in human infections. Infection without scarification did not lead to mortality and evidenced partial self-limiting lesions over the infection kinetics, as also observed in clinical practice [32]. The advantage of HSV-1 ocular infection without scarification has already been shown by Pereira and collaborators, who developed the HSV-1 infection model in BALB/c mice and found that corneal scarification resulted in greater morbidity and mortality rates in this model than in those without scarification [33].

Initially, a different viral inoculum was assessed during infection kinetics to test the experimental model. According to the data from the current study, the percentages of HSV-1 qPCR positive mice and mice presenting some sign of ocular lesion, as well as classification scores, were in accordance with the viral inoculum. Mice infected with 10^9^ PFU/mL had higher values of viral load and developed lesions earlier than those in the group inoculated with 10^7^ PFU/mL, who did not show any lesions. The infection proved to be self-limiting, with viral load decreasing over time due to the latent state of HSV-1. These data reinforced findings by Moein and colleagues, who highlighted that clinical severity (blepharitis, corneal opacity, neovascularization, and epitheliopathy) is more pronounced in mice who are inoculated with a higher load of HSV-1 (2 × 10^6^ PFU/mL) in comparison to the group infected with a lower load (2 × 10^4^ PFU/mL) [34]. These results suggest a load-dependent response and its relevance in understanding the clinical impacts of infection [34]. It is possible that the ocular route of infection, caused by a viral suspension and not by intrastromal injection, as used in our study model, as well as the absence of corneal scarification, contributed to the more pronounced clinical signs observed in the mice infected with the higher viral inoculum (10^9^ PFU/mL). According to published data, C57BL/6 mice infected with 2 × 10^4^ PFU/mL of HSV-1 showed changes in body weight and in ocular lesions when it was intrastromally administered [13]; the same has been reported in mice infected with an ocular inoculum of 1.5×10^6^ PFU/mL directly in the stratified cornea [35]. It is clear that experimental models which avoid manipulations can more closely resemble human infections.

Although antiviral therapies are available to treat HSV-1 infections, none of them can prevent viral reactivation or eliminate the virus from the body [9]. Alternative therapies targeting viral DNA, such as the CRISPR/Cas9 gene editing technology, can be effective. The FDA has approved CRISPR/Cas9 to undergo phase I/II trials, with emphasis on treating human viruses, like HIV, SARS-CoV-2 and Epstein-Barr virus (EBV) (ClinicalTrials.gov, accessed on 10 May 2021: NCT05144386, NCT04990557, NCT03044743, respectively).

In our study, treatments based on a single application of 200 ng/μL CRISPR/Cas9 led to viral load decrease (2 logs, *p* ≤ 0.0001) and decreased the number of mice presenting ocular lesions; the lesion scores also decreased during the infection kinetics in our research. Our research group has already shown the antiviral effect of CRISPR/Cas9 targeting the UL-39 gene of HSV-1 in vitro [21]. This gene is responsible for encoding ribonucleotide reductase, which is a vital enzyme for viral replication [36]. We demonstrated that the sgRNA sequence was inserted into the plasmid (PX459-UL39) for transfection in infected Vero cells in vitro, and based on our qPCR results, the viral load of cells transfected with anti-HSV-1 CRISPR/Cas9 after 48h HSV1 infection, at MOI 0.1 and 0.001 PFU/cell, had decreased by >95% [21]. The CRISPR/Cas9 system has been employed for genomic editing during infections caused by herpes simplex virus (HSV) in vitro, Epstein-Barr virus (EBV), cytomegalovirus (HCMV) and Kaposi’s sarcoma-associated herpesvirus (HHV-8) [22,23]. Van Diemen and colleagues showed powerful HSV-1 replication suppression in human lung fibroblast cells after administering CRISPR/Cas9 lentiviruses to target the number of essential viral genes. Introducing unique gRNAs to target these vital genes has the potential to inhibit viral replication, resulting in an up to 4 logs reduction in HSV-1 titers in our study. However, resistant viral variant development was also observed over time. These variants presented mutations in the sgRNA target site, which made them resistant to subsequent CRISPR/Cas9 editing without compromising virus viability [22]. The efficacy of anti-ICP0 specific small guide RNAs (sgRNAs) targeting HSV-1 was also observed in TC620 human oligodendroglioma cells; it led to an approximately tenfold decrease in infectious virus production. Furthermore, gRNA formulations targeting viral genes have also shown viral replication suppression abilities [37].

The varying effect of anti-HSV-1 CRISPR/Cas9 observed in our studies carried out in vivo, as well as that in other, previous studies, may have been related to the delivery systems for the CRISPR/Cas9 molecules. In our study, CRISPR/Cas9 was applied directly into the eye as suspension; however, other studies have used lentiviral particles as delivery systems [13,22,23,37]. According to the data in the present study, periocular/ocular lesions in the group treated with 200 ng/μL CRISPR/Cas9 were reduced on all dpi, with mice exhibiting a maximum mean value score of 0.12. The study, performed with lentiviral particles containing messenger RNA (mRNA) in vivo, which simultaneously delivered SpCas9 enzyme mRNA and gRNAs targeting viral genes (designated HSV-1 Lentiviral Killer Particles, HELP), revealed a viral load reduction and decreased herpetic keratitis signs and symptoms [13]. The CRISPR/Cas9 anti-HSV-1 dose and therapeutic regimen, as well as those of acyclovir, may be significant variables for different models. Di Yin and colleagues used the acyclovir topical treatment and CRISPR intrastromal injection (1.5 µg) in both mice’s eyes for five days, leading to lesion inhibition; however, only HELP significantly reduced infectious virus production levels [13]. The results in the current research showed that a single application of 200 ng/μL (1 µg) CRISPR/Cas9 suspension was enough to reduce ocular lesions and viral load, i.e., by 2 logs, in mice infected with high viral load (10^9^ PFU/mL) in non-stratified corneas.

The current study showed the efficacy of anti-HSV-1 CRISPR/Cas9 in reducing viral load and periocular/ocular lesions after a single application in a suspension form. This treatment avoided intrastromal injection and used an experimental infection model without corneal scarification. These encouraging findings suggest that CRISPR/Cas9 should be assessed as alternative approach to treat ocular herpes.

## Figures and Tables

**Table 1:** Score classification according to periocular/ocular lesion signs.

Classification	Absent	Mild	Moderate	Intense	Severe
Description	No visible changes	Mild facial edema	Moderate facial edema and periocular/ocular inflammation	Intense facial edema and periocular hair loss	Severe facial edema and periocular/ocular inflammation; extensive hair loss; corneal opacity
Score	0	1	2	3	4

**Table 2:** BALB/c mice infected or not with herpes simplex virus type 1 (HSV-1) and treated or not with CRISPR/Cas9.

Experimental Groups	
G1	Non-infected (NI); negative control
G2	HSV-1 10^7^ PFU/mL and untreated (UNT); positive control
G3	HSV-1 10^9^ PFU/mL and untreated (UNT); positive control
G4	HSV-1 10^9^ PFU/mL and treated CRISPR/Cas9 50 ng/μL
G5	HSV-1 10^9^ PFU/mL and treated CRISPR/Cas9 100 ng/μL
G6	HSV-1 10^9^ PFU/mL and treated CRISPR/Cas9 200 ng/μL

## Data Availability

The original contributions presented in the study are included in the article and Supplementary Materials, further inquiries can be directed to the corresponding author.

## Supplementary Materials

The following supporting information can be downloaded at: https://www.mdpi.com/article/10.3390/biomedicines13071738/s1, Table S1: Guide RNAs selected for the UL39 region. Figure S1: CRISPOR output for gRNA12 and gRNA17 showing no high-confidence off-targets.
