# Supplementary material for: CRISPR/Cas9 Reduces Viral Load in a BALB/c Mouse Model of Ocular Herpes Infection
Source: Biomedicines. 2025 Jul 16;13(7):1738. doi: 10.3390/biomedicines13071738 (PMC12292542; doi:10.3390/biomedicines13071738)
Supplement: Supplementary file 1 [file biomedicines-13-01738-s001.zip › biomedicines-3694376-supplementary.pdf]

Supplementary File

The UL39 region from HSV-1, with 3518 base pairs, was selected, since it is a conserved region and is responsible for encoding the largest subunit of the ribonucleotide reductase enzyme. Using the CRISPOR platform, two guide RNA sequences and the reporter plasmid (green fluorescent protein - GFP) were selected for the UL39 region (Raposo et. al, 2023.).

Table S1: Guide RNAs selected for the *UL39* region.

Guide RNA	Sequence
Guide RNA 12 for *UL39* (sense)	CACCG**CGACGGGAGAGGATGCGGCT**
Guide RNA 12 for *UL39* (antisense)	AAAC**AGCCGCATCCTCTCCCGTCG**C
Guide RNA 17 for *UL39* (sense)	CACCG**CGCTTCGACGGGAGAGGATG**
Guide RNA 17 for *UL39* (antisense)	AAAC**CATCCTCTCCCGTCGAAGCG**C

Source: Raposo et. al, 2023.

Position/ Strand	Guide Sequence + *PAM* + Restriction Enzymes<br>Only G- Only GG- Only A-	MIT Specificity Score	CFD Spec. score	Predicted Efficiency<br>Show all scores<br>Doench '16	Mor.-Mateos	Doench-RuleSet3	Outcome<br>Out-of-Frame	Lindel	Off-targets for 0-1-2-3-4 mismatches + next to PAM	Genome Browser links to matches sorted by CFD off-target score<br>exons only NC_001806.2 only
5 / rev	AGAGGATGCGGCTGGGCGGC *TGG*<br>Enzymes: *Fsp4HI, TauI*<br>Cloning / PCR primers	100	100	32	42	-53	65	72	0 - 0 - 0 - 0 - 0<br>0 - 0 - 0 - 0 - 0<br>0 off-targets	
9 / rev	CGGGAGAGGATGCGGCTGGG *CGG*<br>Enzymes: *BstC8I, Fsp4HI, TauI*<br>Cloning / PCR primers	100	100	62	88	57	63	88	0 - 0 - 0 - 0 - 2<br>0 - 0 - 0 - 0 - 0<br>2 off-targets	4:intergenic:rna0-cds2/cds3<br>4:intergenic:cds59-rna2
12 / rev	CGACGGGAGAGGATGCGGCT *GGG*<br>Enzymes: *Fsp4HI, TauI*<br>Cloning / PCR primers	100	100	53	57	17	68	69	0 - 0 - 0 - 0 - 0<br>0 - 0 - 0 - 0 - 0<br>0 off-targets	
13 / rev	TCGACGGGAGAGGATGCGGC *TGG*<br>Enzymes: *Fsp4HI, TauI*<br>Cloning / PCR primers	100	100	35	51	1	67	62	0 - 0 - 0 - 0 - 0<br>0 - 0 - 0 - 0 - 0<br>0 off-targets	
17 / rev	CGCTTCGACGGGAGAGGATG *CGG*<br>Enzymes: *Fsp4HI, BtsCI, TauI*<br>Cloning / PCR primers	100	100	44	60	-7	62	66	0 - 0 - 0 - 0 - 0<br>0 - 0 - 0 - 0 - 0<br>0 off-targets	

Figure S1: CRISPOR output for gRNA12 and gRNA17 showing no high-confidence off-targets.
Available at: https://crispor.gi.ucsc.edu/crispor.py?batchId=hwew9VP34O1VceL3rYZE
